# «Green-Ligand» in Metallodrugs Design—Cu(II) Complex with Phytic Acid: Synthetic Approach, EPR-Spectroscopy, and Antimycobacterial Activity

**DOI:** 10.3390/molecules30020313

**Published:** 2025-01-15

**Authors:** Kseniya A. Koshenskova, Natalia V. Makarenko, Fedor M. Dolgushin, Dmitriy S. Yambulatov, Olga B. Bekker, Matvey V. Fedin, Sergei A. Dementev, Olesya A. Krumkacheva, Igor L. Eremenko, Irina A. Lutsenko

**Affiliations:** 1 N. S. Kurnakov Institute of General and Inorganic Chemistry, Russian Academy of Sciences, Leninsky Prosp. 31, 119991 Moscow, Russia; ksenia-18.11.99@mail.ru (K.A.K.); yambulatov@yandex.ru (D.S.Y.); ileremenko@yandex.ru (I.L.E.); 2 Institute of Chemistry of the Far Eastern Branch, Russian Academy of Sciences, Prosp. 100-Letiya Vladivostoka, 159, 690022 Vladivostok, Russia; 3 N. I. Vavilov Institute of General Genetics, Russian Academy of Sciences, Gubkina, 3, 119333 Moscow, Russia; 4 International Tomography Center SB RAS, Institutskaya 3a, 630090 Novosibirsk, Russia; mfedin@tomo.nsc.ru (M.V.F.); dementev.s@tomo.nsc.ru (S.A.D.);; 5 Novosibirsk State University, Pirogova 1, 630090 Novosibirsk, Russia; 6 RUDN University, Miklukho-Maklaya 6, 117198 Moscow, Russia

**Keywords:** copper(II) compounds, phytic acid, crystal structure, EPR spectra, antimycobacterial activity

## Abstract

The interaction of sodium phytate hydrate C_6_H_18_O_24_P_6_·xNa·yH_2_O (phytNa) with Cu(OAc)_2_·H_2_O and 1,10-phenanthroline (phen) led to the anionic tetranuclear complex [Cu_4_(H_2_O)_4_(phen)_4_(phyt)]·2Na^+^·2NH_4_^+^·32H_2_O (**1**), the structure of the latter was determined by X-ray diffraction analysis. The phytate **1** is completely deprotonated; six phosphate^−^ fragments (with atoms P1–P6) are characterized by different spatial arrangements relative to the cyclohexane ring (1a5e conformation), which determines two different types of coordination to the complexing agents—P1 and P3, P4, and P6 have monodentate, while P2 and P5 are bidentately bound to Cu^2+^ cations. The molecular structure of the anion complex is stabilized by a set of strong intramolecular hydrogen bonds involving coordinated water molecules. Aromatic systems of phen ligands chelating copper ions participate in strong intramolecular and intermolecular π-π interactions, further contributing to their association. At the supramolecular level, endless stacks are formed, in the voids of which sodium and ammonium cations and water molecules are present. The stability of **1** in the presence of human serum albumin (HSA) was investigated using Electron Paramagnetic Resonance (EPR) spectroscopy. Continuous wave (CW) EPR spectra in water/glycerol frozen solution clearly indicate a presence of an exchange-coupled Cu(II)-Cu(II) dimeric unit, as well as a Cu(II) monomer-like signal arising from spins sufficiently distant from each other, with comparable contributions of two types of signals. In the presence of albumin at a 1:1 ratio (**1** to albumin), the EPR spectrum changes significantly, primarily due to the reduced contribution of the S = 1 fraction showing dipole–dipole splitting. The biological activity of **1** in vitro against the non-pathogenic (model for *Mycobacterium tuberculosis*) strain of *Mycolicibacterium smegmatis* is comparable to the first-line drug for tuberculosis treatment, rifampicin.

## 1. Introduction

The use of natural molecules (including natural cellular metabolites—amino acids, vitamins, lipids, and nucleic acids) to create biologically active complexes is one of the actual directions of bioinorganic, coordination, and medicinal chemistry [1,2,3,4,5,6]. The presence of natural metabolic pathways makes such ligands particularly attractive in the design of compounds with biological activity. Inosithexaphosphoric (phytic) acid (vitamin B8—inositol, IP6, C_6_H_6_[OPO(OH)_2_]_6_) is a natural product («green ligand») of plant origin (phytin is concentrated in the aleurone layer of legume seeds and rice) [7,8]. Inositols, especially myo-inositol and inositol hexakisphosphate, have become increasingly popular recently because of their positive health effects. Inositol pyrophosphate derivatives formed from IP6 increase cellular energy by enhancing glycolysis and mitochondrial function and help to combat metabolic disorders and improve insulin resistance [9]. Inosithexaphosphoric acid has a scientifically proven ability to prevent the development of certain cancer types and metabolic diseases such as type 2 diabetes and nephrolithiasis [10,11,12]. The presence of twelve ionizable protons determines the ability to chelate ions of multivalent metals (Cu, Zn, Fe) [13,14,15,16,17,18]. Phytic acid and its derivatives (phosphoinositols) are found in living cells as part of protein-lipid complexes with a high metabolic rate. This indicates that when complexing with essential metals, for example, copper, the formed complexes will have natural ways of excretion from the cell. The addition of biologically active N-donor ligands, such as 1,10-phenanthroline, increases the stability [19,20] (as a consequence of biological activity) of the compound due to the formation of stacking interactions, increased lipophilicity and changes in physico-chemical properties.

A limited number of inositol-based complexes are presented in the literature—monoligand complexes of copper with phytic acid and 1,10-phenanthroline are known. By changing the molar ratios of phosphoinositol (IP6), the authors [21] obtained three complex compounds [Cu_x_IP6], where x = 1–3. Our first attempts at the complexation of copper(II) with phytic acid and 2,2′-bipyridine (bpy) led to the transformation of the phytate fragment and the formation of a molecular multi-ligand tetra-nuclear complex [(Cu_4_(bpy)_4_(PO_4_)_2_(CO_3_)(H_2_O)_2_]⋅13H_2_O [22]. In this work, during complexation in the {Cu(II)–phyt–phen} system, the synthesis conditions were changed, which resulted in the formation of crystals, the structure of which was determined according to X-ray data. The stability of **1** in the presence of human serum albumin (HSA) was investigated using Electron Paramagnetic Resonance (EPR) spectroscopy. In vitro biological activity was studied in relation to the model non-pathogenic strain *M. smegmatis*.

## 2. Results and Discussion

### 2.1. Synthesis and Characterization

**Scheme 1:**
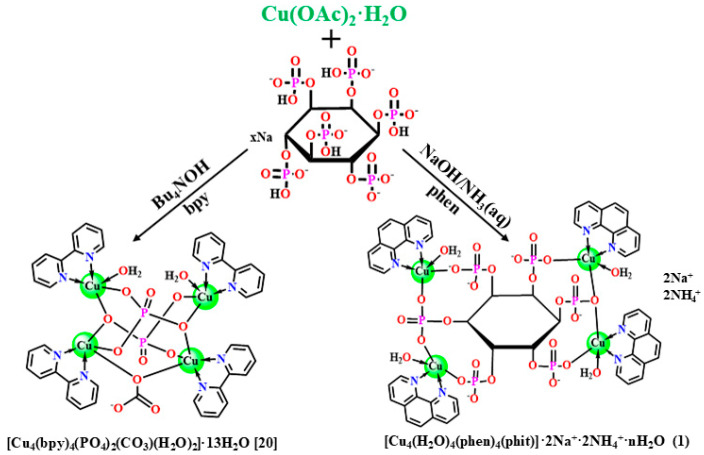
Different paths of forming complexes in the system {Cu(II)–phyt–oligopyridine}.

An earlier experiment showed that the use of Bu_4_NOH base and heating conditions led to the destruction of phytate and the formation of a phosphate-carbonate complex of copper [(Cu_4_(bpy)_4_(PO_4_)_2_(CO_3_)(H_2_O)_2_]⋅13H_2_O [22]. Softening the synthesis conditions and replacing Bu_4_NOH with sodium hydroxide made it possible to keep the phytic fragment unchanged (Scheme 1; see also Experimental Section).

**Scheme 2:**
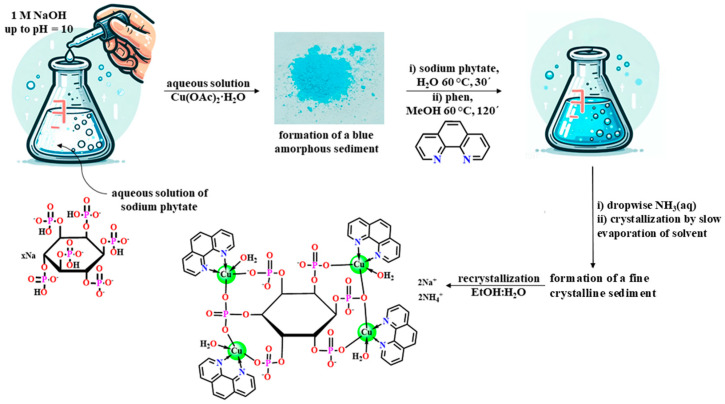
The synthetic experiment path to obtain the complex **1**.

Isolation of an intermediate amorphous product, pH control, changing the reagents ratio and the order of their addition into the reaction mixture, the ammonia solution that promotes better solubility, as well as subsequent recrystallization of the resulting polycrystalline mass from the EtOH:H_2_O mixture, allowed the blue crystals of **1** to be isolated; the molecular structure was determined by the XRD method (Scheme 2).

### 2.2. Crystal Structure of ***1***

**Figure 1:**
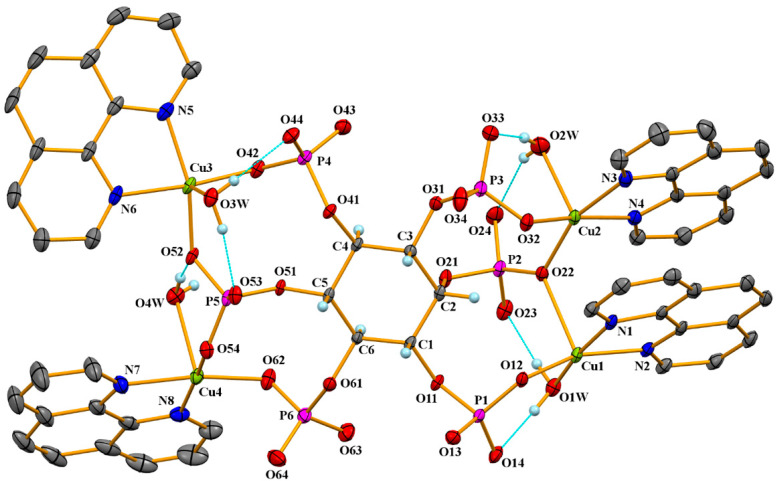
ORTEP representation of the anionic part in the complex **1** with thermal ellipsoids drawn at the 50% probability level. Hydrogen atoms of the phen ligands are omitted for clarity. Intramolecular hydrogen bonds are shown with dotted lines.

The compound **1** crystallizes in the triclinic space group *P*-1 and is a solvated tetra-nuclear ion complex [(Cu_4_(H_2_O)_4_(phen)_4_(phyt)]^4−^⋅2Na^+^·2NH_4_^+^·32H_2_O (Figure 1). The phytate in the structure assumes the most stable conformation in solution up to pH~9, which is characterized by the presence of one axial and five equatorial phosphate groups (1a5e conformation) [23].

Each copper atom in **1** is monodentately bound to two phosphate groups, coordinates a bidentate phenanthroline ligand, and one water molecule. The geometry of the metal polyhedron is a distorted square pyramid {CuN2O3} (copper coordination number CN_Cu_ 5; τ(Cu1) = 0.205, τ(Cu2) = 0.151, τ(Cu3) = 0.329, τ(Cu4) = 0.015; τ = 0 for ideal SQP/τ = 1 for ideal TBP). For three Cu^2+^ ions, Cu2, Cu3, and Cu4, the base of the square-pyramidal coordination environment is determined by two nitrogen atoms of the chelated phen ligand and two oxygen atoms of phosphate groups; the axial position is occupied by oxygen of the water molecule. In the case of the Cu1 ion, the oxygen atom of the coordinated water molecule is located at the base of the square pyramid, and the oxygen atom of the phosphate anion is located in the axial position, which leads to a noticeable redistribution of the length of Cu–O bonds in the coordination environment of copper ions (Table 1).

The phytate anion in **1** is completely deprotonated, which is confirmed by the lengths of the P–O bonds, among which one increased distance (1.605(2)–1.621(2) Å) stands out with the oxygen atom associated with the central six-membered cycle, whereas the lengths of the other three P–O bonds are noticeably shorter and lie in the range 1.505(2)–1.538(2) Å, the highest values among which (1.523(2)–1.538(2) Å) correspond to oxygen atoms bound to copper ions.

All six phosphate groups of phytate are involved in coordination with copper ions: three groups of P1-3 bind ions Cu1 and Cu2, and three groups of P4–6 bind ions Cu3 and Cu4. In both fragments, two phosphate groups (P1 and P3, P4 and P6) are monodentate ligands, bound to only one copper ion. Two more phosphate groups (P2 and P5) perform a bridging function, binding two copper ions (see Table S2). In this case, the P2 group combines Cu1 and Cu2 ions due to simultaneous coordination with the bridging oxygen atom O22, and the P5 group combines Cu3 and Cu4 ions due to their coordination with two different oxygen atoms, O52 and O54, respectively. The difference in the coordination of phosphate groups P2 and P5 is apparently determined by the location relative to the central cyclohexane ring: the P2 group is in the axial position, whereas the P5 group and all other phosphate groups occupy equatorial positions. The molecular structure of the anion complex is stabilized by a set of strong intramolecular hydrogen bonds involving coordinated water molecules (Table S3).

The Cu1 and Cu2 ions bridged by the O22 oxygen atom (the angle Cu1-O22-Cu2 is 112.12(7)°) are brought together at a distance of 3.6298(5) Å. In addition, aromatic systems of phen ligands chelating these copper ions participate in a strong intramolecular π-π interaction (Table S4), further contributing to their association. Note that the formation of a similar binuclear fragment stabilized by intramolecular π-π interactions between polyaromatic ligands occurred in other complexes with the phytate anion, in particular, in the {Cu(II)–phyt–terpy} system Cu...Cu 3.805 Å [24] and in the {Cu(II)–phyt–phen} system Cu...Cu 3.957 Å [25]. Two other copper ions, Cu3 and Cu4, in **1**, bounded by a bridging phosphate group, P5, are located at a distance of 5.4911(5) Å from each other; the planes of their phen ligands are almost perpendicular to each other and widely separated, excluding π-π interaction between them (the dihedral angle between the planes of aromatic fragments is 70.8° and the distance between their centroids is 8.88 Å).

**Figure 2:**
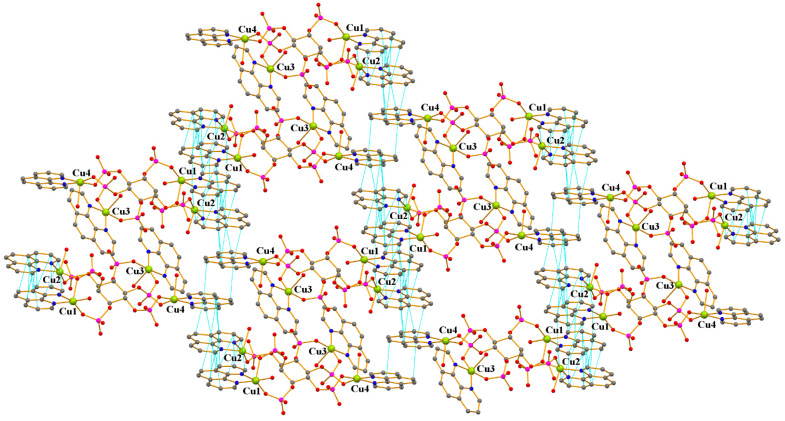
A fragment of the crystal packing of **1** illustrating the formation of stacked supramolecular motifs due to π-π interactions between the phen ligands of neighboring anionic complexes (the shortest intermolecular C…C contacts with a length of 3.313–3.493 Å are shown by dotted lines).

The extended aromatic phen fragments of adjacent anionic complexes in **1** overlap each other, participating in strong π-π interactions and forming the main supramolecular motif of the crystal structure of this compound, namely, infinite stacks located along the (101) direction of the crystal (Figure 2). Large voids forming in the space between the stack fragments are filled in the crystal with sodium and ammonium cations, as well as a large number of molecules of crystallization water (according to X-ray diffraction data, there are 32 water molecules per anion complex). Some of these water molecules form an octahedral coordination environment of sodium cations, and all of them participate in the formation of a complicated system of hydrogen bonds involving ammonium cations and the oxygen atoms of the phosphate groups of the anion.

### 2.3. EPR-Spectroscopy of ***1***

**Figure 3:**
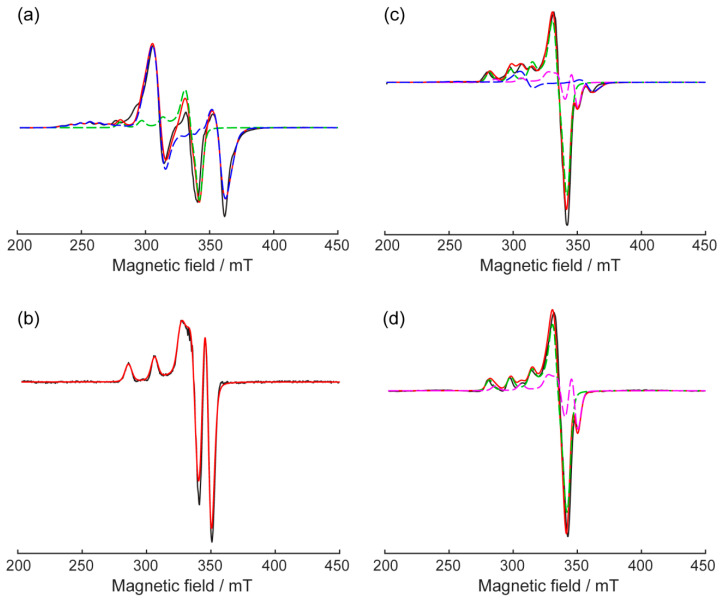
X-band CW EPR spectra of samples recorded at 80 K in a 1:1 mixture of 10 mM PBS buffer and glycerol. Experimental spectra (black noisy lines) were simulated using EasySpin. Dashed colored lines represent simulated spectra for individual fractions (parameters in Table 2); the red line is the final combined simulation weighted as shown in Table 2. (**a**) 1 mM **1**: blue dashed line—tetrameric fraction; green dashed line—monomeric fraction. (**b**) Equimolar complex of 1 mM Cu^2+^ and 1 mM HSA. (**c**) Equimolar complex of 1 mM **1**- and 1 mM HSA: blue dashed line-tetrameric fraction; green dashed line-monomeric fraction; magenta dashed line-Cu^2+^-HSA complex. (**d**) 1:4 complex of 0.25 mM **1**- and 1 mM HSA: green dashed line—monomeric fraction; magenta dashed line—Cu^2+^-HSA complex.

The stability of **1** in the presence of human serum albumin (HSA) was investigated using Electron Paramagnetic Resonance (EPR) spectroscopy. HSA, the most abundant plasma protein, serves as a key transport molecule in the human body, facilitating the delivery of various biomolecules, including drugs, nutrients, and metal ions such as Cu^2+^ and Zn^2+^ [26,27]. Notably, HSA binds metal ions like Cu^2+^ and Zn^2+^ with high affinity [27,28], which can, in some cases, lead to the dissociation of metal-containing complexes [29]. Therefore, assessing the stability of Cu(II)-containing compounds in the presence of HSA is critical for evaluating their potential as therapeutic agents. EPR spectroscopy provides detailed information about the electronic structure, ligand coordination, and magnetic interactions of Cu(II)-containing compounds. To assess the stability of **1**, EPR spectra were recorded for this compound in an aqueous glycerol solution both in the presence and absence of human serum albumin (HSA) (Figure 3a,c,d). Additionally, the EPR spectrum of mononuclear Cu(II) bound to HSA was obtained for comparison (Figure 3b).

Cu(II) ions are characterized by significant g-tensor anisotropy, resulting in complex powder spectra with overlapping lines in the X-band. The interaction of the unpaired copper electron with its nuclear spin (*I* = 3/2), known as hyperfine interaction, splits each spectral line corresponding to a specific orientation into four components. For the experimental data obtained, spectral modeling was performed by EasySpin software [30], accounting for the anisotropy of both the g-tensor and hyperfine interaction tensor (A_Cu(II)_), as well as dipole–dipole interaction between neighboring spins in **1**. The results of this analysis are summarized in Table 2.

The EPR spectrum of **1** in a water-glycerol matrix reveals two distinct fractions (Figure 3a). The dominant fraction, contributing 70% of the signal (shown in blue), exhibits characteristic features of two interacting Cu(II) centers with spins *S*_1_ = 1/2 and *S*_2_ = 1/2, coupled by exchange and dipole–dipole interactions. In a model of total spin *S* = 1, these centers are described by zero-field splitting (ZFS) parameter *D* = 1540 MHz (~55 mT, ~0.05 cm^−1^). Using the point dipole approximation, the measured dipole–dipole coupling corresponds to a Cu(II)-Cu(II) distance of approximately 3.7 Å. This distance likely reflects the separation between the nearest copper ions (Cu-O-Cu link) within the tetramer cluster, and the obtained distance of 3.7 Å is in very good agreement with 3.63 Å between these ions in a crystal. The second fraction, contributing 30% of the spectrum (Figure 3a, green line), has two possible interpretations. Its spectrum resembles that of the monomeric copper species, likely being residual components from the synthesis or some dissolved fraction of the complex. Figure 3a shows a simulation in this model that provides satisfactory agreement with the experiment. However, according to the structure derived from XRD analysis, a second dimeric unit (Cu–O–P–O–Cu) link is present in **1** tetramer, and the Cu…Cu distance in it is larger, being ~5.5 Å. As such, the dipolar splitting between two copper spins might be quite small, providing a monomer-like EPR spectrum. This hypothesis was also tested (see Supporting Information, Figure S1 and Table S1), yielding worse but still reasonable agreement with the experiment. Thus, CW EPR spectra in water/glycerol frozen solution clearly indicate a presence of an exchange-coupled Cu(II)-Cu(II) dimeric unit, as well as a Cu(II) monomer-like signal arising from spins sufficiently distant from each other, with comparable contributions of two types of signals. In the presence of albumin at a 1:1 ratio (**1** to albumin), the EPR spectrum changes significantly, primarily due to the reduced contribution of the *S* = 1 fraction showing dipole–dipole splitting (Figure 3c). To better understand the complex EPR spectrum of **1** in the presence of HSA, we also recorded the spectrum of Cu(II) ions bound to albumin (Figure 3b), which served as a reference for identifying copper ions interacting with the protein. Based on this reference data, the experimental spectrum of **1** in the presence of albumin was well described as a superposition of three distinct fractions: (1) the spectrum of *S* = 1 copper dimer featuring dipole–dipole interactions, shown as a blue dashed line in Figure 3c with a 20% contribution; (2) a fraction corresponding to Cu(II) bound to HSA, contributing 20%; and (3) the predominant fraction, contributing 60%, with g- and A-tensor parameters characteristic of monomeric Cu(II) coordinated by ligands.

At a 4-fold excess of HSA, the *S* = 1 fraction from the original tetramer disappears completely, with the monomeric fraction increasing to 70%, while the remaining 30% correspond to Cu(II) bound to HSA (Figure 3d). While our data do not provide direct evidence of whether the monomeric fraction binds to protein or not, it is clear that the copper coordination environment in the predominant fraction differs from that observed in the binding of mononuclear Cu(II) to HSA.

The g-tensor values for Cu^2+^ ions coordinated by different ligands fall within characteristic ranges, which depend on the ligand environment and the symmetry of the complex. It is also worth noting that the obtained g- and A-tensor values for the monomeric form in the presence of HSA match those reported for copper coordinated by 1,10-phenanthroline [31], which is a component of the original tetrameric complex.

### 2.4. Biological Activity of ***1***

The biological activity of **1** was studied using the nonpathogenic mycobacterial strain *Mycolicibacterium smegmatis*. In mycobacterial strains, the resistance to chemical treatment agents is attributed to the low permeability of its cell wall due to its unusual structure. *M. smegmatis* is a rapidly growing nonpathogenic bacterium, which is therefore used as a model for slowly growing *Mycobacterium tuberculosis* and for the screening of antituberculosis drugs [32]. The *M. smegmatis* test system exhibits higher resistance to antibiotics and antituberculosis agents than *M. tuberculosis*; therefore, the concentration <100 nmol/disk, in contrast to *M. tuberculosis* (<4 µg/mL), is used as the selection criterion [33]. All the results obtained for the in vitro biological activity of the compounds under study were compared with the activity of the first-line antituberculosis drug, Rifampicin (Rif), under the selected conditions of the experiment. The results of the antimycobacterial activity study are presented in Table 3.

As follows from the data presented in Table 3, the activity of the resulting complex exceeds the initial ligands by 7–80 times and is also comparable to the comparison drug rifampicin. The bacterial growth inhibition zone does not overgrow after 120 h, which indicates the bacteriostatic effect of the compound. Comparing with previously obtained phenanthroline complexes of copper(II), an increase in efficiency can be noted when changing the anionic fragment, complex **1** becomes the third in activity and dimeter of the zone of suppression of mycobacterial growth after furoate coordination compounds of copper(II), which is possibly due to its good solubility in water, greater bioavailability.

Thus, new complexes of biogenic metals open up prospects for the creation of more effective antibacterial drugs, which, in turn, can significantly increase the level of treatment of infectious diseases and reduce the development of antibiotic resistance among microorganisms. The most important issue remains the issue of further research aimed at optimizing the structure and their interaction with biological targets.

## 3. Materials and Methods

### 3.1. General Remarks

Commercial reagents and solvents were used for the synthesis: Cu(OAc)_2_·H_2_O (95%, Acros), sodium phytate hydrate C_6_H_18_O_24_P_6_·xNa·yH_2_O (Sigma-Aldrich, St. Louis, MO, USA), 1,10-phenanthroline (99% Alfa Aesar, Haverhill, MA, USA), sodium hydroxide NaOH, aqueous ammonia solution (20%), methanol (≥99%), and ethanol (95%). The IR spectra were recorded on a Perkin Elmer Spectrum 65 spectrophotometer equipped with a Quest ATR Accessory (Specac, England) by the attenuated total reflectance (ATR) in the range of 400–4000 cm^−1^ (see Supporting Information, Figures S2 and S3). Microprobe analyses were carried out using a Carlo Erba EA 1108 Series CHNS Elemental Analyzer (Center of Collective Use of IGIC RAS).

X-ray diffraction data for **1** were collected at 100 K with a Bruker SMART APEXII diffractometer, using graphite monochromated Mo-Kα radiation (λ = 0.71073 Å, ω-scans). The crystal data and structure refinement parameters are given in Table 4. Semiempirical absorption correction based on equivalent reflections was applied using the SADABS [37] program. The structure was solved by direct methods and refined by the full-matrix least-squares technique against *F*^2^ with the anisotropic thermal parameters for all non-hydrogen atoms using the SHELXL [38] program package.

A large number of residual electron density peaks are localized in an asymmetric unit, some of which are defined as positions for two sodium cations (one of which is in a special position at an inversion center, while the other position is half occupied) and two ammonium cations, as well as numerous partially disordered water molecules. In the final stage of refinement, a model with 22 crystallization water molecules was used. The contribution of highly disordered crystallization water molecules (approximately 10 molecules) was excluded using the SQUEEZE routine implemented in the PLATON program [39]. Thus, according to the X-ray diffraction data, compound **1** contains two sodium cations, two ammonium cations, and 32 water molecules per anionic complex. Hydrogen atoms of the coordinated water molecules, ammonium cations, and well-resolved crystallization water molecules were localized from Fourier syntheses; the rest of the hydrogen atoms were placed in calculated positions, and they all were refined in the isotropic approximation within the riding model. CCDC 2407213 contains the supplementary crystallographic data for this manuscript. These data can be obtained free of charge from the Cambridge Crystallographic Data Centre http://www.ccdc.cam.ac.uk, accessed on 4 January 2025.

Sample preparation for EPR: A 12.5 mM solution of **1** in 10 mM PBS buffer was prepared and stored at –20 °C. This stock solution was diluted to the required concentrations for the experiments. Human serum albumin (HSA) was purchased from Sigma-Aldrich (catalog no. A3782, St. Louis, MO, USA). To study the EPR spectrum of **1**, the 12.5 mM stock solution was diluted with 10 mM PBS to a concentration of 2 mM. Then, 20 μL of this diluted solution was mixed with 20 μL of glycerol, resulting in a final concentration of 1 mM **1** in a 1:1 mixture of 10 mM PBS buffer and glycerol. For investigating changes in the EPR spectra of **1** when complexed with HSA, the 12.5 mM **1** stock solution was diluted to 2 mM with 10 mM PBS. HSA was added to achieve a final concentration of 2 mM for both **1** and HSA. Then, 20 μL of this mixture was combined with 20 μL of glycerol, forming an equimolar complex of 1 mM **1** and 1 mM HSA in a 1:1 mixture of 10 mM PBS buffer and glycerol. To prepare a 1:4 **1**–HSA complex, the **1** stock solution was diluted to 0.5 mM, while HSA concentration was maintained at 2 mM. The same mixing steps were followed, resulting in a final complex with 0.25 mM **1** and 1 mM HSA in a 1:1 mixture of 10 mM PBS buffer and glycerol. To obtain a reference spectrum of the Cu^2+^-HSA complex, a 20 μL solution of 2 mM CuSO_4_ was prepared, and HSA was added to achieve a final concentration of 2 mM. The mixture was then combined with 20 μL of glycerol, resulting in an equimolar complex containing 1 mM Cu^2+^ and 1 mM HSA. For EPR measurements, samples had a total volume of 40 μL and were placed into quartz tubes (outer diameter: 4 mm; inner diameter: 2.5 mm).

EPR measurements. CW EPR experiments were conducted using a Bruker ELEXSYS E580 spectrometer at the Center of Collective Use “mass spectrometric investigations” SB RAS (X-band 9.69–9.71 GHz). Prior to placement in the spectrometer cavity, samples were shock-frozen in liquid nitrogen. The temperature of all measurements was T = 80 K.

General experimental settings were as follows: a central magnetic field of 345.0 mT, a sweep width of 300 mT, a microwave power of 0.196 mW, a modulation frequency of 100 kHz, a modulation amplitude of 0.5 mT, and 1024 data points were collected. Sample-specific parameters were adjusted based on the composition of each sample. The number of scans ranged from 4 to 7, and the conversion times ranged from 327.68 ms to 655.36 ms. Experimental EPR spectra were simulated using the ’pepper’ function from EasySpin 6.0.6. This function calculates field-swept and frequency-swept solid-state CW EPR spectra. The system parameters obtained from the simulations are presented in Table 2.

### 3.2. Synthesis of ***1***

A sample of sodium phytinate hydrate salt, C_6_H_18_O_24_P_6_·xNa·yH_2_O (0.165 g, 0.25 mmol), was dissolved in 10 mL of H_2_O, then 1 M of sodium hydroxide NaOH solution to pH = 10. Cu(OAc)_2_·H_2_O (0.3 g, 1.5 mmol) was dissolved in a minimum amount of distilled water and added to an alkaline solution of sodium phytinate. An amorphous blue precipitate was immediately observed, which was separated by decantation, washed with water, and dried in air to a constant mass. An aqueous solution (20 mL) of sodium phytinate hydrate C_6_H_18_O_24_P_6_·xNa·yH_2_O (0.165 g, 0.25 mmol) was added to the resulting dry sediment. The resulting reaction mixture was left to react at 60 °C for 30 min. Then, 1,10-phenanthroline (0.27 g, 1.5 mmol) dissolved in 5 mL of MeOH was added to the resulting solution. The reaction mixture was kept at 60 °C for 120 min, and an aqueous ammonia solution was added drop by drop until a clear blue solution was obtained, which was filtered and allowed to slowly crystallize. After some time, a fine crystalline precipitate was formed, which was recrystallized from an EtOH:H_2_O mixture to obtain crystals suitable for X-ray diffraction. The yield was 0.45 g (55%). Anal. calc. C_54_H_98_Cu_4_N_10_O_50_Na_2_P_6_: C 29.84, H 4.55, N 6.45 Found: C 30.06, H 4.63, N 4.58. FT-IR (ATR, ν/cm^−1^): 3380 br.w, 3199 br.w, 3053 m, 2925 w, 2884 w, 2808 m, 2697 br.w, 2618 br.w, 1719 w, 1662 w, 1593 v.s, 1574 v.s, 1514 s, 1426 s, 1406 s, 1364 m, 1310 s, 1218 m, 1151 m, 1103 m, 1053 m, 967 m, 916 w, 858 v.s, 794 s, 768 s, 720 v.s, 646 m, 562 m, 506 m, 428 m, 412 m.

## 4. Conclusions

A new anionic copper(II) complex based on the natural ligand sodium phytate and 1,10-phenanthroline has been synthesized. According to X-ray diffraction data, four Cu^2+^ cations are mono- and bidentately bound via six phosphate fragments to the cyclohexane ring, and differences in their binding are determined by the spatial non-equivalence of PO_4_^3-^ in the cycle. The supramolecular level is characterized by the formation of oligopyridine stacks held by π-stacking interactions. The resulting voids contain sodium, ammonium ions, and water molecules. The EPR experiments performed showed that at a 4-fold excess of HSA, the S = 1 fraction of the initial tetramer completely disappears, and the monomeric fraction increases to 70%, with the remaining 30% corresponding to Cu(II) bound to HSA. Although our data do not provide direct evidence of whether the monomeric fraction binds to the protein or not, it is clear that the copper environment in the predominant fraction differs from that observed when mononuclear Cu(II) binds to HSA. The MIC values obtained from the action of complex **1** on the smegmatis strain are comparable to the action of the reference drug rifampicin.

## Figures and Tables

**Table 1:** Selected bond lengths (d, Å) in **1**.

	Cu1	Cu2	Cu3	Cu4
Cu–N	2.006(2), 2.012(2)	2.025(2), 2.033(2)	2.039(2), 2.021(2)	2.025(2), 2.030(2)
Cu–O_(OPO3)_	1.9163(17)	1.9259(17)	1.9277(17)	1.9175(18)
Cu–O_(O-bridge)_ Cu–O(_OPO-bridge)_	2.3921(17)	1.9740(17)	1.9475(17)	1.9434(18)
Cu–O_(water)_	1.9697(18)	2.2681(19)	2.2704(18)	2.3460(19)

**Table 2:** Parameters from EPR spectra modeling using EasySpin [30]: g- and A-tensors, ZFS parameters D and E.

Sample	Fraction	Weight	g-Tensor [g_xx_, g_yy_, g_zz_]	A-Tensor [A_xx_, A_yy_, A_zz_], MHz	D-Tensor [D, E], MHz
1	Monomer	30%	[2.064, 2.064, 2.272]	[40, 40, 520]	-
	Tetramer	70%	[2.066, 2.076, 2.272]	[80, 150, 470]	[1540, 60]
1-HSA (1:1)	HSA-Cu(II)	20%	[2.037, 2.066, 2.190]	[20, 60, 621]	-
	Tetramer	20%	[2.066, 2.076, 2.272]	[80, 150, 470]	[1540, 60]
	Monomer	60%	[2.054, 2.078, 2.261]	[40, 40, 525]	-
1-HSA (1:4)	HSA-Cu(II)	30%	[2.037, 2.066, 2.190]	[20, 60, 621]	-
	Monomer	70%	[2.054, 2.078, 2.261]	[40, 40, 525]	-
HSA-Cu(II)	HSA-Cu(II)	100%	[2.037, 2.066, 2.190]	[20, 60, 621]	-

**Table 3:** Results in vitro antibacterial activity of **1** against *M. smegmatis*.

Compound	MIC, nmol/disk	Zone of Inhibition, mm		Ref.
	24 h	24 h	120 h	
**1**	6	6.7 ± 0.3	6.6 ± 0.1 *	this work
[(Cu_4_(bpy)_4_(PO_4_)_2_(CO_3_)(H_2_O)_2_]⋅13H_2_O	50	6.6 ± 0.2	6.4 ± 0.2 *	[22]
[Cu(2fur)_2_(phen)]	5	7 ± 0.5	7 ± 0.5 *	[34]
[Cu(benz)_2_phen]	12.5	6.5 ± 0.2	6.4 ± 0.1 *	[34]
[Cu(Hpz)_2_(OOCCF_3_)_2_phen]	10	6.8 ± 0.5	6.5 ± 0.1	[35]
[Cu(Hpz)_2_(Otf)_2_phen]	10	6.5 ± 0.1	6.4 ± 0.1	[35]
[Cu(Hpz)_2_Clphen]Cl	8	6.6 ± 0.1	6.5 ± 0.1	[35]
[Cu_2_(nfur)_4_(nphen)_2_]	5	6.5 ± 0.5	6.5 ± 0.2 *	[36]
[Cu_2_(2fur)_4_(nphen)_2_]·MeOH	10	7.0 ± 0.5	6.4 ± 0.2 *	[36]
C_6_H_18_O_24_P_6_·xNa·yH_2_O	>500	0	0	this work
phen	45	7.5 ± 0.5	0	this work
Rif	6	7.2 ± 0.3 *	7.13 ± 0.35 *	this work

The diameter of the paper disk is 6 mm. * The zone of growth inhibition of *M. smegmatis* is transparent; there is no weak background growth of the culture (bactericidal effect). 2fur, nfur, benz,—anions 2-furoic-, 5-nitro-2-furoic- and 3-Cl-benzoic acids, respectively. Hpz—pirazole; nphen—5-nitro-1,10-phenantroline.

**Table 4:** Crystal data and structure refinement for **1**.

Parameter	Value
Empirical formula	C_54_H_118_Cu_4_N_10_Na_2_O_60_P_6_
Formula weight	2353.54
Crystal size (mm)	0.28 × 0.12 × 0.06
Temperature (K)	100
Crystal system	triclinic
Space group	*P*-1
a (Å)	16.8441 (11)
b (Å)	17.8246 (12)
c (Å)	17.9297 (12)
α (deg)	71.620 (2)
β (deg)	72.861 (2)
γ (deg)	64.024 (2)
V (Å3)	4514.5 (5)
Z	2
dcalc (g·cm^−3^)	1.731
μ (cm^−1^)	11.63
Tmin/Tmax	0.644/0.933
2θmax (deg)	58
no. of unique reflns (Rint)	23974 (0.0555)
no. of obsd reflns (I > 2σ(I))	20312
R_1_ (on *F* for obsd reflns)	0.0438
*w*R_2_ (on *F*^2^ for all reflns)	0.1220
GOOF	1.026
diff. peak/hole (e/Å^−3^)	1.445/−1.113

## Data Availability

The structure parameters of the obtained compound were deposited into the Cambridge Structural Database (CCDC 2407213) deposit@ccdc.cam.ac.uk or http://www.ccdc.cam.ac.uk/data_request/cif (accessed on 3 January 2025).

## Supplementary Materials

The following supporting information can be downloaded at https://www.mdpi.com/article/10.3390/molecules30020313/s1, Figure S1: X-band CW EPR spectra of 1 mM N6 recorded at 80 K in a 1:1 mixture of 10 mM PBS buffer and glycerol; Figure S2: IR spectra of 1; Figure S3: Superimposition of IR spectra of 1, sodium phytate, and 1,10-phenanthroline; Table S1: Parameters from EPR spectra modeling using EasySpin assuming two contributions with different ZFS parameters D and E; Table S2: Selected bond lengths (Å) and angles (deg.) for 1; Table S3: Parameters of H-bonds in **1**; Table S4: Parameters of intra- and intermolecular π-π interactions in **1**.
