# Supplementary material for: «Green-Ligand» in Metallodrugs Design—Cu(II) Complex with Phytic Acid: Synthetic Approach, EPR-Spectroscopy, and Antimycobacterial Activity
Source: Molecules. 2025 Jan 15;30(2):313. doi: 10.3390/molecules30020313 (PMC11767443; doi:10.3390/molecules30020313)
Supplement: Supplementary file 1 [file molecules-30-00313-s001.zip › molecules-3394545-supplementary.pdf]

molecules

MDPI

Supplementary materials for the article

«Green-Ligand» in Metallodrugs Design – Cu(II) Complex with Phytic Acid: Synthetic approach, EPR-spectroscopy and Anti-mycobacterial activity

Kseniya A. Koshenskova [1], Natalia V. Makarenko [2*], Fedor M. Dolgushin [1], Dmitriy S. Yambulatov [1], Olga B. Bekker [3], Matvey V. Fedin [4,5], S.A. Dementev [4,5], O.A Krumkacheva [4,5], Igor L. Eremenko [1], Irina A. Lutsenko [1,6]**

[1] N. S. Kurnakov Institute of General and Inorganic Chemistry, Russian Academy of Sciences, 31 Leninsky prosp., 119991 Moscow, Russian Federation. Fax: +7 (495) 955 4817.
[2] Institute of Chemistry of the Far Eastern Branch, Russian Academy of Sciences, Prosp. 100-Letiya Vladivostoka, 159, 690022 Vladivostok, Russia
[3] N. I. Vavilov Institute of General Genetics, Russian Academy of Sciences, Gubkina, 3, 119333, Moscow, Russia
[4] International Tomography Center SB RAS, Institutskaya str. 3a, 630090 Novosibirsk, Russia
[5] Novosibirsk State University, Pirogova 1, 630090, Novosibirsk, Russia
[6] RUDN University, Miklukho-Maklaya St. 6, 117198, Moscow, Russia

* Correspondence: makarenko@ich.dvo.ru (N. V. M.);
irinalu05@rambler.ru (I. A. L.)

Citation: To be added by editorial staff during production.

Academic Editor: Firstname Lastname

Received: date
Revised: date
Accepted: date
Published: date

Copyright: © 2023 by the authors. Submitted for possible open access publication under the terms and conditions of the Creative Commons Attribution (CC BY) license (https://creativecommons.org/licenses/by/4.0/).

S1. Auxiliary EPR simulation data

An alternative hypothesis to explain the shape of the EPR spectrum of N6 dissolved in water/glycerol relies on assumption that the central signal refers to the second dimeric unit in N6 (Cu-O-P-O-Cu) unit. In this case we have to assume rather weak dipole-dipole interactions between two copper spins. The obtained value used in simulation in Fig.1S is D = 300 MHz, which corresponds to the spin-spin distance of ca. 6.4 A in point dipole approximation. This value is slightly larger than that obtained from single-crystal diffraction data (5.5 A), but still represent a reasonable scenario.

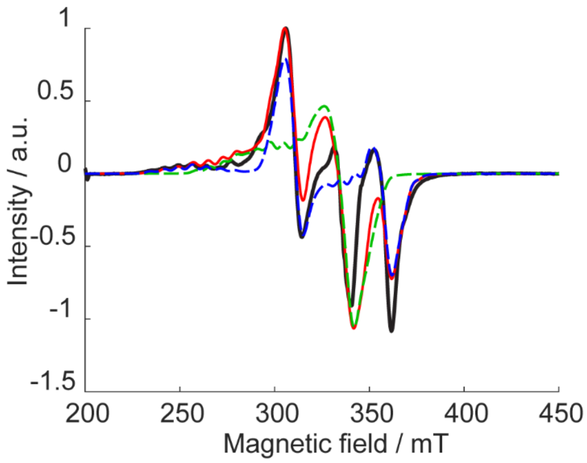

Figure S1. X-band CW EPR spectra of 1 mM N6 recorded at 80 K in a 1:1 mixture of 10 mM PBS buffer and glycerol. Experimental spectra (black noisy lines) were simulated using EasySpin. Dashed colored lines represent simulated spectra for individual fractions (parameters in Table 2); the red line is the final combined simulation weighted as shown

in Table S1: blue dashed line—tetrameric fraction with D = 1540 MHz; green dashed line— tetrameric fraction with D = 300 MHz.

Table S1. Parameters from EPR spectra modeling using EasySpin assuming two contributions with different ZFS parameters D and E.

**Sample**	**Fraction**	**Weight**	**g-tensor [gxx,gyy,gzz]**	**A-tensor [Axx,Ayy,Azz], MHz**	**D-tensor [D, E], MHz**
**1**	Dimeric unit I (Cu-O-Cu)	50%	[2.040, 2.040, 2.40]	[80, 160, 470]	[300, 70]
	Dimeric unit II (Cu-O-P-O-Cu)	50%	[2.066, 2.076, 2.272]	[80, 150, 470]	[1540, 60]

S2. Supporting X-ray data

Table S2. Selected bond lengths (Å) and angles (deg.) for **1**.

Bond		Bond	
Cu(1)-O(12)	1.9163(17)	P(3)-O(33)	1.510(2)
Cu(1)-O(1W)	1.9697(18)	P(3)-O(34)	1.521(2)
Cu(1)-N(1)	2.006(2)	P(3)-O(32)	1.5271(17)
Cu(1)-N(2)	2.0121(19)	P(3)-O(31)	1.6170(18)
Cu(1)-O(22)	2.3921(17)	P(4)-O(43)	1.5124(19)
Cu(2)-O(32)	1.9259(17)	P(4)-O(44)	1.5150(18)
Cu(2)-O(22)	1.9740(17)	P(4)-O(42)	1.5383(18)
Cu(2)-N(3)	2.025(2)	P(4)-O(41)	1.6102(17)
Cu(2)-N(4)	2.033(2)	P(5)-O(53)	1.5084(18)
Cu(2)-O(2W)	2.2681(19)	P(5)-O(54)	1.5234(18)
Cu(3)-O(42)	1.9277(17)	P(5)-O(52)	1.5378(17)
Cu(3)-O(52)	1.9475(17)	P(5)-O(51)	1.6048(16)
Cu(3)-N(6)	2.021(2)	P(6)-O(64)	1.505(2)
Cu(3)-N(5)	2.039(2)	P(6)-O(63)	1.5104(19)
Cu(3)-O(3W)	2.2704(18)	P(6)-O(62)	1.538(2)
Cu(4)-O(62)	1.9175(18)	P(6)-O(61)	1.6167(18)
Cu(4)-O(54)	1.9434(18)	O(11)-C(1)	1.423(3)
Cu(4)-N(7)	2.025(2)	O(21)-C(2)	1.436(3)
Cu(4)-N(8)	2.030(2)	O(31)-C(3)	1.429(3)
Cu(4)-O(4W)	2.3460(19)	O(41)-C(4)	1.431(3)
P(1)-O(14)	1.5124(18)	O(51)-C(5)	1.428(3)
P(1)-O(13)	1.5166(18)	O(61)-C(6)	1.430(3)
P(1)-O(12)	1.5298(18)	C(1)-C(2)	1.525(3)
P(1)-O(11)	1.6208(17)	C(1)-C(6)	1.530(3)
P(2)-O(24)	1.5113(19)	C(2)-C(3)	1.525(3)

P(2)-O(23)	1.5163(19)	C(3)-C(4)	1.523(3)
P(2)-O(22)	1.5376(17)	C(4)-C(5)	1.525(3)
P(2)-O(21)	1.6173(17)	C(5)-C(6)	1.524(3)

Angle		Angle	
O(12)-Cu(1)-O(1W)	96.12(8)	O(33)-P(3)-O(31)	102.91(11)
O(12)-Cu(1)-N(1)	91.88(8)	O(34)-P(3)-O(31)	107.69(10)
O(1W)-Cu(1)-N(1)	171.24(8)	O(32)-P(3)-O(31)	107.82(10)
O(12)-Cu(1)-N(2)	158.92(8)	O(43)-P(4)-O(44)	115.59(11)
O(1W)-Cu(1)-N(2)	89.43(8)	O(43)-P(4)-O(42)	109.74(10)
N(1)-Cu(1)-N(2)	81.83(8)	O(44)-P(4)-O(42)	112.64(10)
O(12)-Cu(1)-O(22)	99.05(6)	O(43)-P(4)-O(41)	109.66(9)
O(1W)-Cu(1)-O(22)	93.14(7)	O(44)-P(4)-O(41)	100.47(9)
N(1)-Cu(1)-O(22)	89.12(7)	O(42)-P(4)-O(41)	108.14(10)
N(2)-Cu(1)-O(22)	100.93(7)	O(53)-P(5)-O(54)	111.83(10)
O(32)-Cu(2)-O(22)	93.05(7)	O(53)-P(5)-O(52)	114.11(10)
O(32)-Cu(2)-N(3)	92.37(8)	O(54)-P(5)-O(52)	108.69(10)
O(22)-Cu(2)-N(3)	160.08(8)	O(53)-P(5)-O(51)	109.57(9)
O(32)-Cu(2)-N(4)	169.13(8)	O(54)-P(5)-O(51)	109.19(9)
O(22)-Cu(2)-N(4)	90.50(8)	O(52)-P(5)-O(51)	103.03(9)
N(3)-Cu(2)-N(4)	80.97(9)	O(64)-P(6)-O(63)	115.06(11)
O(32)-Cu(2)-O(2W)	96.36(8)	O(64)-P(6)-O(62)	112.95(12)
O(22)-Cu(2)-O(2W)	103.58(7)	O(63)-P(6)-O(62)	109.08(11)
N(3)-Cu(2)-O(2W)	94.83(8)	O(64)-P(6)-O(61)	102.75(11)
N(4)-Cu(2)-O(2W)	92.78(8)	O(63)-P(6)-O(61)	108.45(10)
O(42)-Cu(3)-O(52)	94.88(7)	O(62)-P(6)-O(61)	108.09(10)
O(42)-Cu(3)-N(6)	174.22(8)	C(1)-O(11)-P(1)	119.26(14)
O(52)-Cu(3)-N(6)	89.82(8)	P(1)-O(12)-Cu(1)	127.19(11)
O(42)-Cu(3)-N(5)	93.22(8)	C(2)-O(21)-P(2)	124.62(14)
O(52)-Cu(3)-N(5)	154.47(8)	P(2)-O(22)-Cu(2)	126.42(10)
N(6)-Cu(3)-N(5)	81.12(9)	P(2)-O(22)-Cu(1)	121.45(9)
O(42)-Cu(3)-O(3W)	94.98(7)	Cu(2)-O(22)-Cu(1)	112.12(7)
O(52)-Cu(3)-O(3W)	98.87(7)	C(3)-O(31)-P(3)	120.22(15)
N(6)-Cu(3)-O(3W)	87.61(8)	P(3)-O(32)-Cu(2)	128.50(11)
N(5)-Cu(3)-O(3W)	104.50(8)	C(4)-O(41)-P(4)	124.81(14)
O(62)-Cu(4)-O(54)	94.79(8)	P(4)-O(42)-Cu(3)	128.46(10)
O(62)-Cu(4)-N(7)	170.55(9)	C(5)-O(51)-P(5)	122.45(14)
O(54)-Cu(4)-N(7)	92.24(8)	P(5)-O(52)-Cu(3)	126.03(10)
O(62)-Cu(4)-N(8)	91.03(9)	P(5)-O(54)-Cu(4)	128.68(11)
O(54)-Cu(4)-N(8)	169.67(8)	C(6)-O(61)-P(6)	122.40(15)
N(7)-Cu(4)-N(8)	81.11(9)	P(6)-O(62)-Cu(4)	121.34(12)
O(62)-Cu(4)-O(4W)	100.97(8)	O(11)-C(1)-C(2)	110.44(17)

O(54)-Cu(4)-O(4W)	92.79(7)	O(11)-C(1)-C(6)	108.72(18)
N(7)-Cu(4)-O(4W)	84.96(8)	C(2)-C(1)-C(6)	111.32(18)
N(8)-Cu(4)-O(4W)	94.47(8)	O(21)-C(2)-C(3)	108.96(18)
O(14)-P(1)-O(13)	115.51(11)	O(21)-C(2)-C(1)	108.67(18)
O(14)-P(1)-O(12)	112.77(10)	C(3)-C(2)-C(1)	109.73(17)
O(13)-P(1)-O(12)	110.23(10)	O(31)-C(3)-C(4)	107.68(18)
O(14)-P(1)-O(11)	102.18(10)	O(31)-C(3)-C(2)	111.08(18)
O(13)-P(1)-O(11)	107.87(9)	C(4)-C(3)-C(2)	111.46(18)
O(12)-P(1)-O(11)	107.58(9)	O(41)-C(4)-C(3)	107.52(18)
O(24)-P(2)-O(23)	113.84(11)	O(41)-C(4)-C(5)	107.32(17)
O(24)-P(2)-O(22)	113.39(11)	C(3)-C(4)-C(5)	110.44(18)
O(23)-P(2)-O(22)	110.12(10)	O(51)-C(5)-C(6)	109.60(18)
O(24)-P(2)-O(21)	104.52(10)	O(51)-C(5)-C(4)	107.83(17)
O(23)-P(2)-O(21)	106.15(10)	C(6)-C(5)-C(4)	113.41(17)
O(22)-P(2)-O(21)	108.26(9)	O(61)-C(6)-C(5)	107.56(17)
O(33)-P(3)-O(34)	115.15(13)	O(61)-C(6)-C(1)	108.78(18)
O(33)-P(3)-O(32)	112.32(11)	C(5)-C(6)-C(1)	108.21(18)
O(34)-P(3)-O(32)	110.34(11)		

Table S3. Parameters of H-bonds in **1**.

D-H...A	d(D-H)	d(H...A)	d(D...A)	<(DHA)
O(1W)-H(1WA)...O(23)	0.86	1.75	2.603(3)	170
O(1W)-H(1WB)...O(14)	0.83	1.85	2.667(3)	166
O(2W)-H(2WA)...O(33)	0.73	2.03	2.736(3)	162
O(2W)-H(2WB)...O(24)	0.72	2.07	2.749(3)	157
O(3W)-H(3WA)...O(44)	0.75	1.97	2.706(3)	169
O(3W)-H(3WB)...O(53)	0.89	1.86	2.734(3)	168
O(4W)-H(4WA)...O(20W)	0.71	2.11	2.807(3)	169
O(4W)-H(4WB)...O(52)	0.91	1.86	2.761(3)	167
N(9)-H(9A)...O(13)#2	0.91	1.90	2.810(4)	171
N(9)-H(9B)...O(34)	0.94	1.91	2.847(3)	171
N(9)-H(9C)...O(25W)	0.83	2.00	2.823(4)	173
N(9)-H(9D)...O(44)	0.88	2.04	2.904(3)	168
O(5W)-H(5WB)...O(20W)#3	0.97	1.84	2.791(4)	164
O(10W)-H(10A)...O(26W)	0.84	2.00	2.776(7)	155
O(10W)-H(10B)...O(23W)	0.97	1.85	2.766(7)	157
N(10)-H(10C)...O(33)	0.85	1.88	2.729(4)	178
N(10)-H(10D)...O(43)	0.84	1.91	2.748(4)	176
N(10)-H(10F)...O(14W)#2	0.90	1.97	2.819(6)	156
O(11W)-H(11B)...O(64)	1.00	1.70	2.659(4)	161
O(12W)-H(12A)...O(14)	0.83	1.93	2.758(4)	178

O(12W)-H(12B)...O(63)	0.89	1.84	2.720(4)	171
O(13W)-H(13A)...O(64)	0.85	2.41	3.187(5)	153
O(13W)-H(13B)...O(13)	0.95	1.86	2.785(4)	167
O(7W)-H(7WB)...O(22W)	0.99	1.81	2.719(4)	152
O(14W)-H(14B)...O(24W)	0.87	2.11	2.950(7)	161
O(8W)-H(8WA)...O(43)	0.81	2.03	2.811(3)	162
O(15W)-H(15A)...O(64)	0.88	1.66	2.520(8)	164
O(15W)-H(15B)...O(15'')	0.88	1.88	2.678(11)	149
O(15')-H(15C)...O(54)	0.87	2.40	2.933(9)	120
O(15')-H(15D)...O(64)	0.87	1.94	2.791(10)	164
O(15'')-H(15F)...O(25W)	0.87	2.47	3.283(6)	156
O(15'')-H(15F)...O(53)	0.87	2.28	2.829(4)	121
O(8W)-H(8WB)...O(24)	0.80	2.13	2.902(4)	162
O(16W)-H(16A)...O(15')	0.89	1.85	2.698(14)	160
O(16W)-H(16B)...O(17')	0.89	1.92	2.674(12)	142
O(16W)-H(16B)...O(33)#2	0.89	2.49	3.191(13)	137
O(17')-H(17C)...O(33)#2	0.87	2.11	2.736(7)	129
O(9W)-H(9WB)...O(9W)#3	0.85	2.33	2.815(5)	117
O(19W)-H(19A)...O(11W)	0.87	2.06	2.835(9)	147
O(20W)-H(20A)...O(22W)	0.90	1.80	2.677(4)	164
O(21W)-H(21A)...O(44)	0.86	1.89	2.726(4)	164
O(22W)-H(22A)...O(42)	0.81	1.96	2.772(4)	171
O(22W)-H(22B)...O(9W)#3	0.84	2.10	2.838(5)	147
O(24W)-H(24A)...O(13)	0.87	1.97	2.722(4)	144
O(24W)-H(24B)...O(11W)#1	0.85	2.23	2.740(5)	119
O(25W)-H(25A)...O(34)#2	0.75	2.05	2.791(3)	172
O(25W)-H(25B)...O(53)	0.84	2.05	2.882(3)	172
O(26W)-H(26A)...O(43)	0.95	1.79	2.727(4)	166
O(26W)-H(26B)...O(7W)	0.93	1.87	2.789(5)	172

Symmetry transformations used to generate equivalent atoms:

#1 -x+1,-y+2,-z+1 #2 -x+2,-y+2,-z+1 #3 -x+2,-y+2,-z

Table S4. Parameters of intra- and intermolecular π-π interactions in **1**.

Cg(I)	Cg(J)	Symmetry operator	Cg-Cg, Å	ω, deg.[b)]	Slippage, Å[c)]
Intramolecular interactions					
Cg5	Cg7	x,y,z	3.8702(18)	4.31(13)	1.795
Cg6	Cg8	x,y,z	3.4345(17)	3.82(13)	0.946
Cg8	Cg14	x,y,z	3.6335(14)	3.51(13)	1.417
Cg14	Cg15	x,y,z	3.5944(17)	3.63(13)	1.082
Intermolecular interactions					
Cg5	Cg6	2-x,1-y,1-z	3.5732(17)	2.64(13)	1.337
Cg6	Cg14	2-x,1-y,1-z	3.6762(16)	1.32(13)	1.537
Cg7	Cg11	1+x,-1+y,z	3.784(2)	8.66(17)	1.322
Cg8	Cg17	1+x,-1+y,z	3.7173(19)	7.78(14)	1.349
Cg9	Cg10	2-x,3-y,-z	3.7279(17)	2.41(15)	1.558
Cg9	Cg16	2-x,3-y,-z	3.549(2)	1.43(15)	1.035
Cg11	Cg15	-1+x,1+y,z	3.791(2)	7.62(16)	1.587
Cg12	Cg17	1-x,3-y,-z	3.7284(18)	1.93(15)	1.487
Cg15	Cg17	1+x,-1+y,z	3.6437(17)	7.53(14)	1.134
Cg17	Cg17	1-x,3-y,-z	3.9412(17)	0.00(15)	1.974

[a)] Cg(I) – centroid of 6-membered ring, were (5) is N1,C7,C8,C9,C10,C11 ring; (6) is N2,C12,C13,C14,C15,C16 ring; (7) is N3,C19,C20,C21,C22,C23 ring; (8) is N4,C24,C25,C26,C27,C28 ring; (9) is N5,C31,C32,C33,C34,C35; (10) is N6,C36,C37,C38,C39,C40 ring; (11) is N7,C43,C44,C45,C46,C47 ring; (12) is N8,C48,C49,C50,C51,C52 ring; (14) is C10,C11,C12,C13,C18,C17 ring; (15) is C22,C23,C24,C25,C30,C29 ring; (17) is C46,C47,C48,C49,C54,C53 ring;

[b)] ω – dihedral angle between planes of the (I) and (J) 6-membered rings;

[c)] Slippage – distance between Cg(I) and perpendicular projection of Cg(J) on ring (I).

S3. Supporting IR spectra data

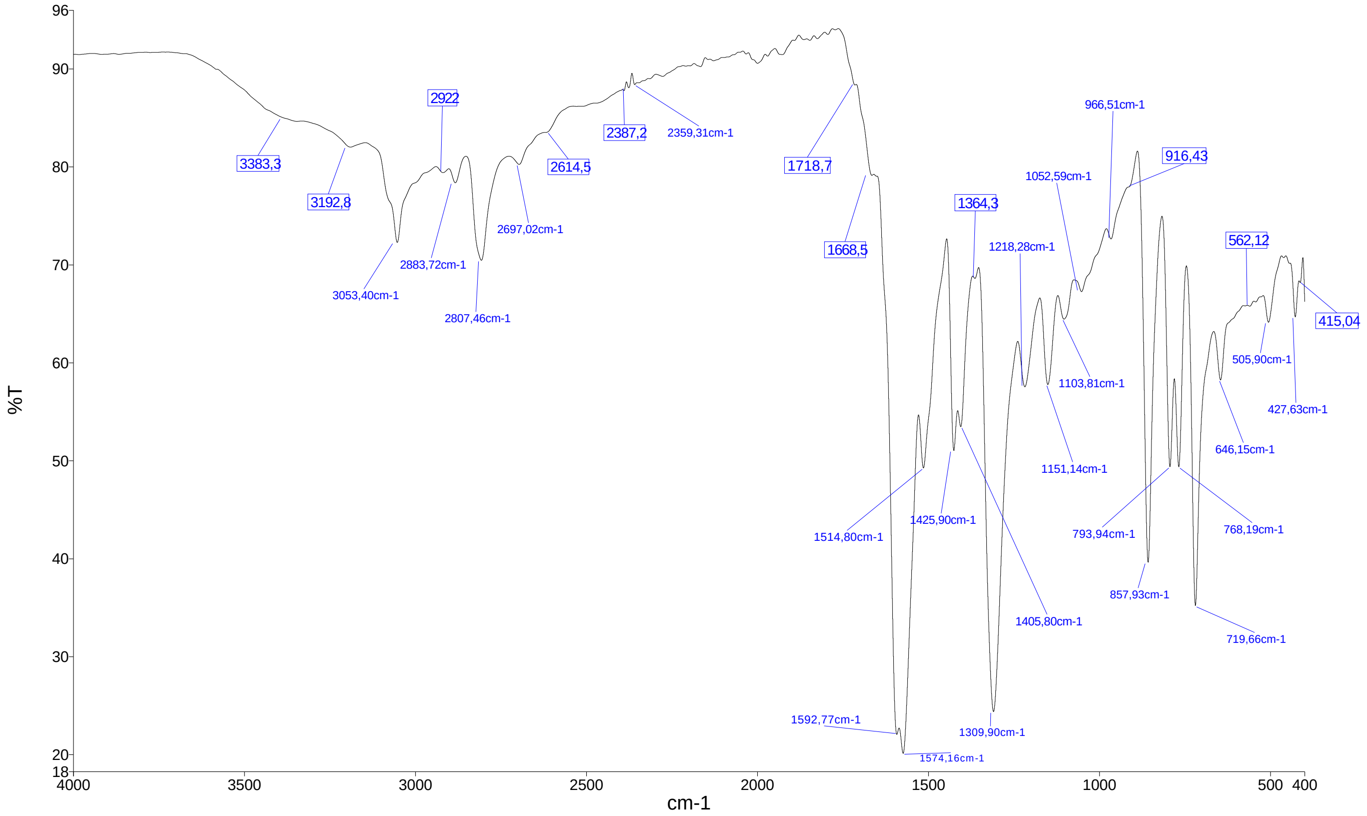

Figure S2. IR-spectra of **1**.

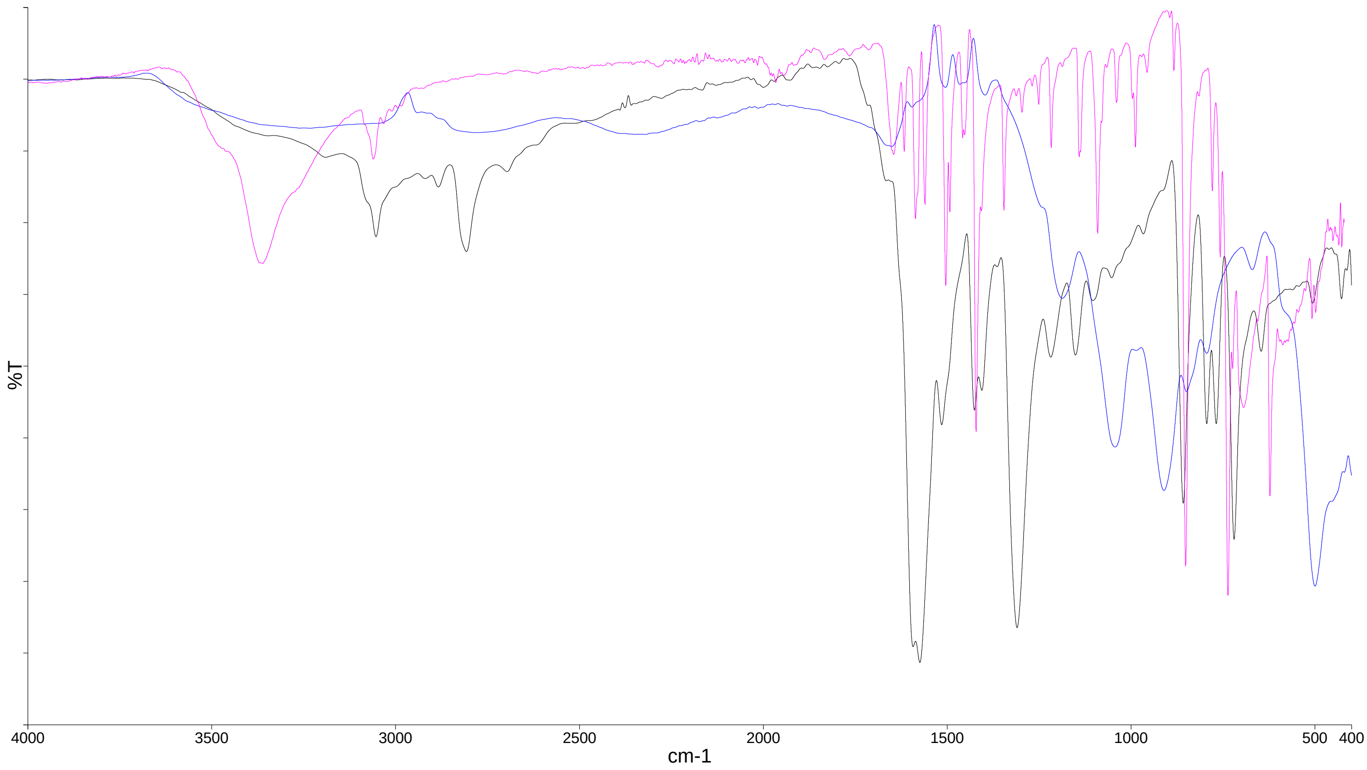

Figure S3. Superimposition of IR spectra of **1** (black), sodium phytate (blue) and 1,10-phenanthroline (pink).
